# mRNA-based vaccine technology for HIV

**DOI:** 10.15190/d.2022.9

**Published:** 2022-06-30

**Authors:** Andra Fortner, Octavian Bucur

**Affiliations:** ^1^Albert-Ludwigs-Universitat Freiburg, Germany; ^2^Victor Babes National Institute of Pathology, Bucharest, Romania; ^3^Viron Molecular Medicine Institute, Boston, MA 02108, USA

**Keywords:** HIV, AIDS, antiretroviral therapy, vaccine, bNAbs, mRNA.

## Abstract

Human immunodeficiency virus (HIV) poses a major health problem around the globe, resulting in hundred-thousands of deaths from AIDS and over a million new infections annually. Although the standard treatment of HIV infection, antiretroviral therapy, has proven effective in preventing HIV transmission, it is unsuitable for worldwide use due to its substantial costs and frequent adverse effects. Besides promoting HIV/AIDS awareness through education, there is hardly an alternative for inhibiting the spread of the disease. One promising approach is the development of an HIV vaccine. Unfortunately, the high variability of envelope proteins from HIV subtypes, their frequency of mutation and the lack of fully understanding the mechanisms of protection against the virus constitute an obstacle for vaccine development. Efforts for developing successful anti-HIV vaccines have been underway for decades now, with little success. Lately, significant progress has been made in adopting the novel mRNA vaccine approach as an anti-HIV strategy. mRNA vaccines received a great thrust during the COVID-19 pandemic. Now, several mRNA-based HIV vaccines are undergoing clinical trials to evaluate their safety and efficacy. This review offers an overview of the pathogenesis and treatment of HIV / AIDS, previous efforts of HIV vaccine development and introduces mRNA vaccines as a promising and potential game changing platform for HIV vaccination.

## SUMMARY

*1. Introduction*

*2. HIV*

- *2.1 Introduction*

- *2.2 HIV replication cycle*

- *2.3 Transmission and pathogenesis*

- *2.4 AIDS*

- *2.5 Current treatment and prevention methods *

*3. HIV vaccines*

*4. mRNA-based vaccines*

- *4.1 What do mRNA vaccines consist of?*

- *4.2 How do they work?*

- *4.3 Approaches of mRNA vaccines*

*5. mRNA-based vaccines for HIV*

*6. Benefits and limitations of the anti-HIV mRNA-based vaccines*

*7. Conclusion*

## 1. Introduction

Acquired immunodeficiency syndrome (AIDS), the disease resulting from infection with the human immunodeficiency virus (HIV), is the second most fatal infectious disease, leading to numerous deaths annually and posing a major health problem, especially in Sub-Saharan African countries^1^. According to the World Health Organization (WHO), 680.000 people died from AIDS and 1,5 million people got infected with HIV in 2020^2^. In the US, approximately 1,2 million people are currently living with HIV/AIDS and approximately 34.800 people acquired the virus in 2019^3^.

HIV is a virus of the retroviridae family that attacks the immune system, leading to an increased susceptibility of the organism to diseases and thus, causes AIDS. Although treatment consisting of lifelong administration of antiretroviral medication is effective in prolonging patients’ freedom of symptoms in the latent period and in impeding transmission, the infection with HIV cannot be cured^4-6^. Furthermore, antiretroviral therapy implies great costs^7,8^ and adherence of the patient to the therapy plan is essential. In order to prevent further spread of HIV / AIDS infection, it has been the focus of research to find an efficient HIV vaccine.

However, due to the complex viral features of HIV, this is a very challenging task. The international renown of mRNA-based vaccines during the COVID-19 pandemic encourages the use of this novel platform for developing and delivering complex vaccination formulas.

This article provides an overview over HIV infection and introduces mRNA vaccines and their potential in HIV vaccine development.

## 2. HIV

### 2.1 Introduction

**Figure 1:**
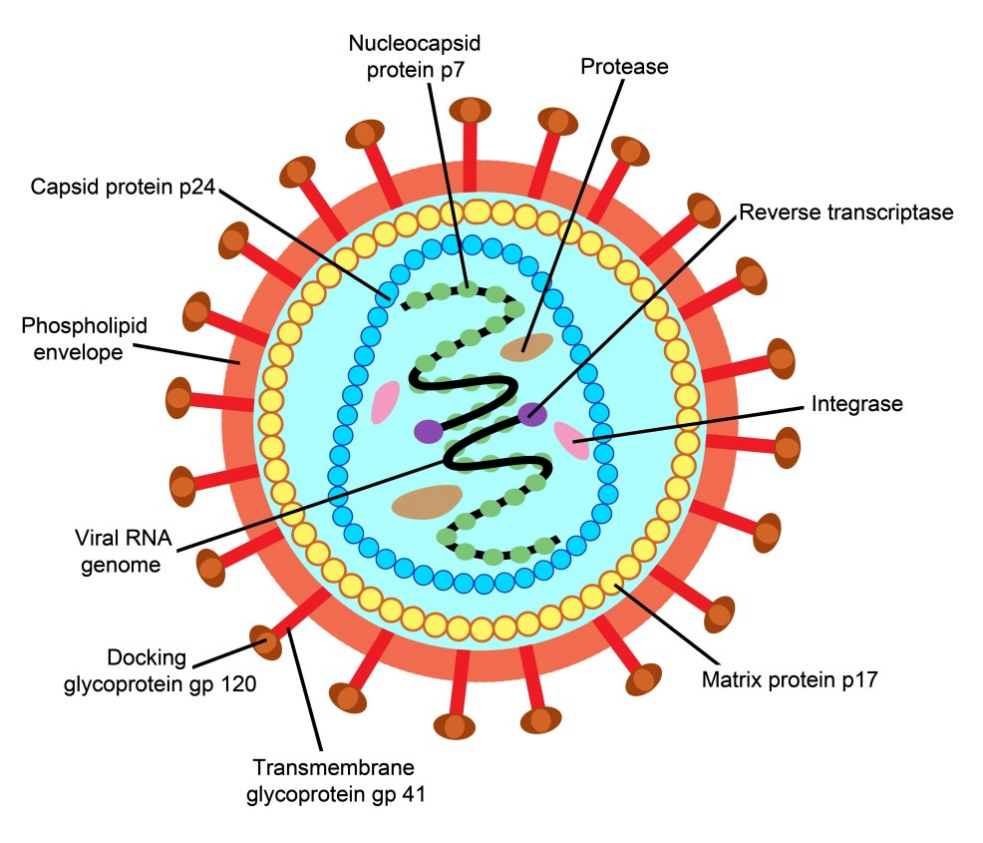
HIV structure HIV contains an RNA genome and the enzymes reverse transcriptase, integrase and HIV protease, that are all surrounded by a capsid, matrix proteins and an envelope. Acquired from Alamy (alamy.de website^11^).

Human immunodeficiency virus (HIV) is the cause of AIDS. Currently, two types of HIV can be distinguished: HIV-1 and HIV-2^9^. HIV-1 can be found worldwide, whereas HIV-2 is the predominant type in Western Africa^10^. The virus measures 100 nm in diameter and is made up of an RNA-genome surrounded by a capsule^9,10^. The RNA is single-stranded and consists of two identical copies that - amongst others - contain the genes gag, pol and env, that encode important viral proteins and are typical for retroviruses^10^. While the gag gene codes for structural core and matrix proteins (p24, p7, p6, p17), the env gene encodes the glycoproteins gp120 and gp41 found in the viral envelope that are used to bind to the CD4 receptor of the host cell^9^. Moreover, the pol gene carries the information for different enzymes needed for the HIV replication cycle: reverse transcriptase, integrase and HIV protease (Figure 1).

### 2.2 HIV replication cycle

The virus uses seven steps to replicate:

#### 2.2.1 Binding and entry

With the help of the envelope protein gp120, HIV can attach itself to cells expressing the CD4 receptor on their surface, namely immune cells, such as the T-helper-lymphocytes in the blood or progenitors in the bone marrow^9,12^. This induces a change in conformation of gp120, revealing an HIV-coreceptor recognizing chemokines^9^. These are small molecules involved in stimulating the migration of leukocytes during inflammation processes. While there are several types of chemokine receptors, in most cases CXCR4 and CXCR5 function as coreceptors for HIV^12^. When the viral protein gp120 is simultaneously bound to the CD4 and chemokine receptor, cell entry is attained through gp41-mediated membrane fusion^9^.

#### 2.2.2 Uncoating

This word describes the release of a viral genome into a host cell’s cytoplasm.

#### 2.2.3 Reverse transcription

Once being inside the host, retroviruses face several problems that they solve in a very interesting way. One of them is that the viral and host genome are incompatible as they differ in the nucleic acid used, making integration of the viral into the host genome impossible. This is where the viral enzymes come into play: reverse transcriptase possesses the ability to convert RNA into complementary DNA (cDNA)^13^. This process takes place in the cytoplasm and begins with the synthesis of an RNA/DNA hybrid double helix^13^. Later, the RNA strand is degraded through ribonuclease H activity and is replaced with the second complementary DNA strand^13^. The cDNA can also be referred to as provirus^13^.

#### 2.2.4 Integration of the provirus

Another aspect refers to the integration of the viral genome into the host genome. The viral cDNA must first be transported into the host nucleus and integrated into the host genome in order to transcribe an mRNA strand the host cell’s ribosomes can read to produce the proteins needed for viral replication. The enzyme responsible for these purposes is called integrase^14^. Firstly, it removes several nucleotides from the 3’ ends of the cDNA, thus leaving sticky ends of the provirus (3’ processing reaction)^14^. In this state the provirus can enter the nucleus. Here, integrase cuts the host DNA and connects viral DNA 3’-OH groups with DNA 5’-phosphates (strand transfer)^14^. This leaves a gap between the viral 5’ end and the host DNA, which is repaired by the host cell’s machinery^14^. Once it is integrated into the host genome, the provirus can remain dormant for years before becoming activated^15^.

#### 2.2.5 Synthesis of viral proteins

If the infected host cell is activated, the provirus can be transcribed into mRNA^15^. In an initial phase, regulatory proteins are synthesized from the mRNA, namely Tat and Rev, which enhance transcription of HIV-genes^9^. Hence, longer RNA transcripts can be produced in the next phase, that encode the information for structural HIV proteins and are synthesized in the cytoplasm using the host cell machinery^9^. Interestingly, larger proteins are often produced in a first step, just to be divided into separate viral particles^9^. For example, a synthesized 160 kD precursor molecule is later cleaved into the pol and gag proteins by HIV-specific protease^9^.

#### 2.2.6 Assembly

During assembly, a new virion is formed by enclosing two viral mRNA strands and the enzymes needed for replication within the HIV capsid made up of HIV core proteins^9^.

#### 2.2.7 Budding

The virion can now cross the plasma membrane towards the extracellular space, receiving a lipid envelope in the process^16^.

The newly released HIV-viruses can continue infecting other CD4+ cells, namely T-helper cells, monocytes, macrophages and dendritic cells^9^.

**Figure 2:**
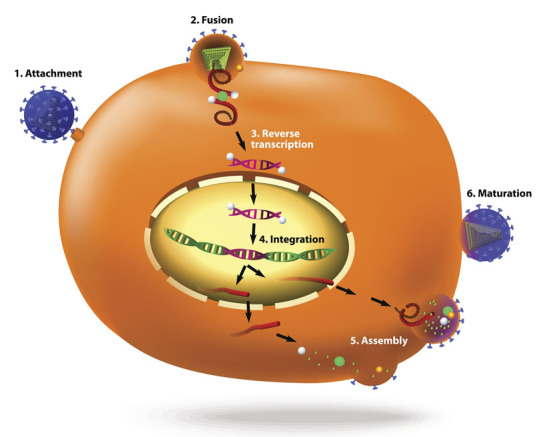
HIV replication cycle After attaching its g120 to the CD4+ receptor and chemokine coreceptor of a host cell (1), viral entry and uncoating takes place (2). The viral enzyme reverse transcriptase turns the viral RNA genome into a cDNA strand (3) which can enter the nucleus in order to be integrated into the DNA-genome of the host cell by action of the viral enzyme integrase (4). Now, biosynthesis of the viral genome can take place creating HIV-RNA strands and viral proteins that can assemble (5) and leave the cell by budding (6) in a next step. Acquired from istockphoto.com^17^.

Replication steps are detailed in Figure 2.

### 2.3 Transmission and pathogenesis

Following transmission of HIV, the course of the disease can be divided into three stages: acute phase, chronic/asymptomatic phase and terminal pahse/AIDS.

HIV transmission can occur in different ways. The most common transmission events are sexual intercourse, exchange of body fluids such as blood, e.g. by sharing needles or syringes for drug injection, and perinatal transmission^18^. According to the UNAIDS report from 2020, 65% of HIV infections worldwide were observed in key populations that are relatively often exposed to the above-mentioned transmission mechanisms, such as sex workers and drug addicts^19^. Hence, HIV infection necessitates direct contact of infected blood cells from the HIV-infected person with skin damage or abrasions existent in the host person^9^. Whether HIV is transmitted or not depends on the contagiousness of the HIV subtype, its concentration in the infectious fluid transmitted, the infection route and in the susceptibility of the host person^9,20^. Furthermore, studies suggest that the prevalence of other infectious diseases in the host that are sexually transmitted, e.g. herpes simplex types 2 and 1 producing genital ulcers, bacterial vaginosis etc, increases the risk of HIV infection^12,21^.

#### 2.3.1 Acute phase

**Figure 3:**
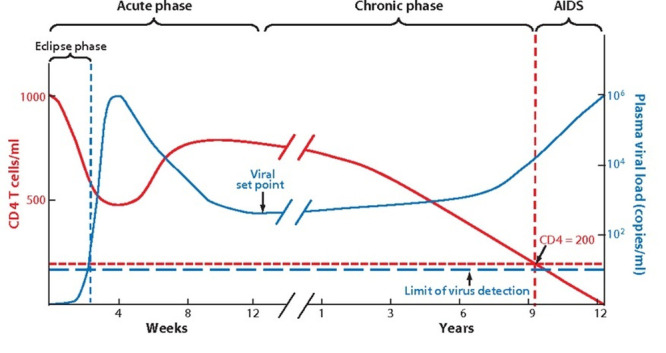
The course of HIV infection The diagram shows the changes of CD4 cell count (red) and plasma viral load (blue, amount of HIV detectable in blood) per milliliter in the course of HIV disease. Three stages can be distinguished. 1. The acute phase starts with the eclipse phase, where the virus cannot yet be detected. After this threshold is surpassed, characteristics of this phase are a peak in plasma viral load, while CD4+ cell numbers drop and then partially recover. 2. In the asymptomatic phase, the body relatively manages to keep a set point of viral load with ongoing immune reactions and CD4+ cell depletion. 3. The terminal phase, also called AIDS, is defined by a CD4+ cell drop below 200 cells per µL blood, clinical symptoms become apparent and opportunistic infections lead to a fatal outcome. Reproduced from the reference^30^(distributed under the “Creative Commons CC0 License”)

After acquiring HIV, the virus starts its replication in the mucosa and submucosa, which causes an innate and adaptive immune response^20^. This is characterized by cytokine and chemokine secretion, and propagation of the infection to dendritic cells and CD4+ lymphocytes that can carry HIV to the lymphatic tissue^12^. Infected immune cells are either eliminated or they persist as viral reservoirs, meaning that they carry the dormant provirus in their genome until reactivation of the virus occurs^9,22^. Hence, viral load inside the body dramatically increases, whereas CD4+ lymphocyte numbers significantly drop during the early acute phase^12^. HIV becomes detectable in the blood 1 - 3 weeks after infection, once the amount of free viral RNA and p24 antigen, an HIV core protein, have sufficiently increased^20^. This event marks the end of the eclipse phase^20^ (see Figure 3). HIV copies in the blood peak at about 4 weeks after infection with over 1 million copies per ml^23^. However, viral load quickly regresses due to the host’s immune response, which is accompanied by seroconversion, describing the onset of antibody production, and a partial recovery in CD4+ cell number^12^. Seroconversion usually takes place within 3 to 5 weeks after infection and can be detected by analyzing antibodies in blood samples^24^. At the end of the acute phase, a setpoint in viral and CD4+ cells is reached^24^. Possible symptoms during this period resemble those of an influenza infection and include headache, fever and rashes^24^. Studies have found correlations between the severity of symptoms during acute phase infection and of the prognosis of HIV patients^9,25^.

#### 2.3.2 Chronic, asymptomatic phase (clinical latency)

The second phase of HIV infection is characterized by an asymptomatic period in which HIV continuously multiplies and stimulates the humoral and cell-mediated immune response accompanied by inflammation, while gradually depleting the organism of its CD4+ immune cells^12^. On the one hand, antibodies against HIV antigens produced by B-cells aim to specifically bind and neutralize free HI viruses, as well as to mark HIV for Antibody-dependent Cellular Cytotoxicity (ADCC), a process in which the antibody-tagged virus can be recognized by natural killer cells and other immune cells^26^. On the other hand, viral antigens that are being expressed on major histocompatibility complex (MHC) proteins of infected CD4+ cells can also be detected by cytotoxic T-cells, triggering the release of perforins and granzymes and thus, the induction of apoptosis in the infected CD4+ cell^27^. Along comes the secretion of cytokines, mediators of inflammation, further increasing immune activation^21^. However, all of these measures do not achieve HIV eradication because of the remaining viral reservoirs and high mutation rate^21^. As the disease progresses, the lymphatic tissue gets damaged, and the CD4+ lymphocyte number progressively declines^9^. The duration of this phase extends from 2-3 years to over a decade depending on the HIV subtype, i.e. the severity of escape mutations, and the vulnerability of the host^21^. In conclusion, the immune system’s attempt to control the viral infection results in an immune system exhaustion and dysfunction.

#### 2.3.3 AIDS

The last phase is acquired immunodeficiency syndrome (AIDS), which is characterized by the proceeding viral replication and a drop in CD4+ lymphocyte number below 200 CD4+ T cells per µL blood^9,24,28^ (normal: 500 - 1500 cells / µL)^28^. At this stage, the immune system has already been too severely compromised to maintain protective function from pathogens, thus, making the organism vulnerable to opportunistic infections with bacteria, other viruses, fungi, parasites or cancer which can quickly become lethal^9,28^.

#### 2.3.4 Elite controllers

HIV-infected people that are not treated with ART but nevertheless manage to maintain undetectable viral loads for one year or more are referred to as Elite controllers^29^. These people are very rare and account for less than 1% of the HIV-infected. Differences in MHC proteins and other receptors of various immune cells and their response to infection have been investigated, but the underlying mechanisms that explain the virological control are still unknown. The phases of HIV infection are detailed in Figure 3.

### 2.4 Current treatment and prevention methods

#### Antiretroviral therapy (ART)

HIV patients are treated with drug combinations that target different steps of the HIV replication process. This method has proved to be highly effective being able to slow down the progression of the disease, but often comes along with side effects and requires the patient’s strict and life-long adherence to the therapy plan to avoid mutations resulting in irreversible drug resistance of the virus^31^. It should be pointed out that HIV / AIDS can only be suppressed, not cured, due to the escape of the dormant provirus from the immune system. Nevertheless, viral load can be reduced to a great extent using this therapy, limiting the risk of mutation and prolonging life expectancy^32^.

Currently, five classes of ART medication are being used.

· **entry inhibitors^32^**

These substances prevent attachment of the virus to a host cell by binding to HIV envelope proteins.

· **nucleoside reverse transcription inhibitors (NRTIs)^32^**

As soon as these substances get integrated into the cDNA during synthesis by the enzyme reverse transcriptase, they lead to chain termination.

· **non-nucleoside reverse transcription inhibitors (NNRTIs)^32^**

These drugs use a specific binding site of the enzyme reverse transcriptase and inactivate it through non-competitive inhibition.

· **integrase strand transfer inhibitors (INSTIs)**

These substances hinder the integration of the cDNA into the host genome by binding to the enzyme integrase.

· **protease inhibitors (PIs)^33^**

By blocking the HIV protease, these substances impede viral assembly.

The usage of ART is not limited to treating infected individuals but has also proven to be effective in preventing HIV transmission in the context of pre- and post-exposure prophylaxis^5,12^. Despite this, the application of ART as a prevention method is complicated and impractical because of the problem of establishing broad availability to the drug and the substantial costs that arise^5^. That’s why a lot of hope relies in the development of a potent vaccine to ensure protection from HIV among the public.

## 3. HIV vaccines

Although a lot of research has been conducted to develop an HIV vaccine, for years none have proven to be effective because of many challenging features of HIV. Those include the huge variability of HIV antigens in the viral envelope, which may differ up to 35%, and the frequent escape mutations occuring in the virus^34,35^. During the last years, research has focused on broadly neutralizing antibodies (bNAbs) as the key substance, and recently, vaccines using the nucleic acid mRNA are getting more and more attention.

### 3.1 Broadly neutralizing antibodies (bNAbs)-based vaccines

In the course of the disease, 20 - 30 % of individuals naturally produce bNAbs 2 - 4 years post-infection^5^. HIV bNAbs are antibodies that are able to effectively neutralize commonly circulating HIV subtypes by binding to highly conserved areas of the virus^36^. More precisely, these antibodies bind to antigens on the HIV Env protein, a protein needed for attachment of the virus to the host cell’s CD4 receptor^5,36^. Env consists of the glycoprotein gp120, interacting with the CD4 receptor, and gp41, anchoring Env in the viral envelope^5,36^. Simultaneously, with their constant region, bNAbs are able to bind to the Fc receptor on immune cells, also stimulating inflammatory processes and cytotoxicity^37^. Thus, the goal is to induce bNAbs against target epitopes of HIV in the infected organism in order to enhance effective immunity. Many approaches have extensively been studied. Here, we briefly present three of them that have shown potential and have progressed in the last years.

#### 3.1.1 Env-resembling molecule

One idea is the injection of a molecule that structurally resembles the Env protein, into the host organism to elicit an immune response with bNAb-production. A major challenge of this approach is the difficulty in reproducing the hidden nature of conserved epitopes of Env. Namely, such epitopes are mostly masked by glycans and its accessibility seems to vary depending on the state the flexible Env currently is in^34,38^. The challenge resides in finding a simultaneously stable and immunogenic substance^39^. After extensive research, some advances have been made. For instance, BG505.SOSIP.664 gp140 is such a protein mimicking clade A Env protein that has shown first success and is currently undergoing phase 1 of clinical trials (NCT03699241)^39-41^.

#### 3.1.2 Nanoparticles for germline-targeting

Another idea is the usage of HIV nanoparticles for germline-targeting^39,42^. This means that nanoparticles designed to be similar to the site, Env gp120 binds to the CD4 receptor with, aim to activate rare B-cell precursors that undergo the complex affinity maturation in order to synthesize bNAbs^5^. Preclinical studies of the nanoparticle eOD-GT8 provided promising results, i.e. bNAb production from B-cell precursors of the class VRC01 in mice^5,39^. Also, recently the results of the phase 1 clinical trial (NCT03547245) revealed that VRC01-bNAbs could be found in 97% of the vaccinated participants^43,44^.

#### 3.1.3 B-cell lineage vaccine

An alternative method is the design of a B-cell lineage vaccine. This method involves multiple injections, starting with an initial form of Env in the first injection followed by gradually evolved Env proteins in the next injections, thus mimicking its evolutionary progression which triggers B-cells into production of bNAbs^39^. In addition, this approach is in phase 1 of clinical trials (NCT03220724)^45^.

**Figure 4:**
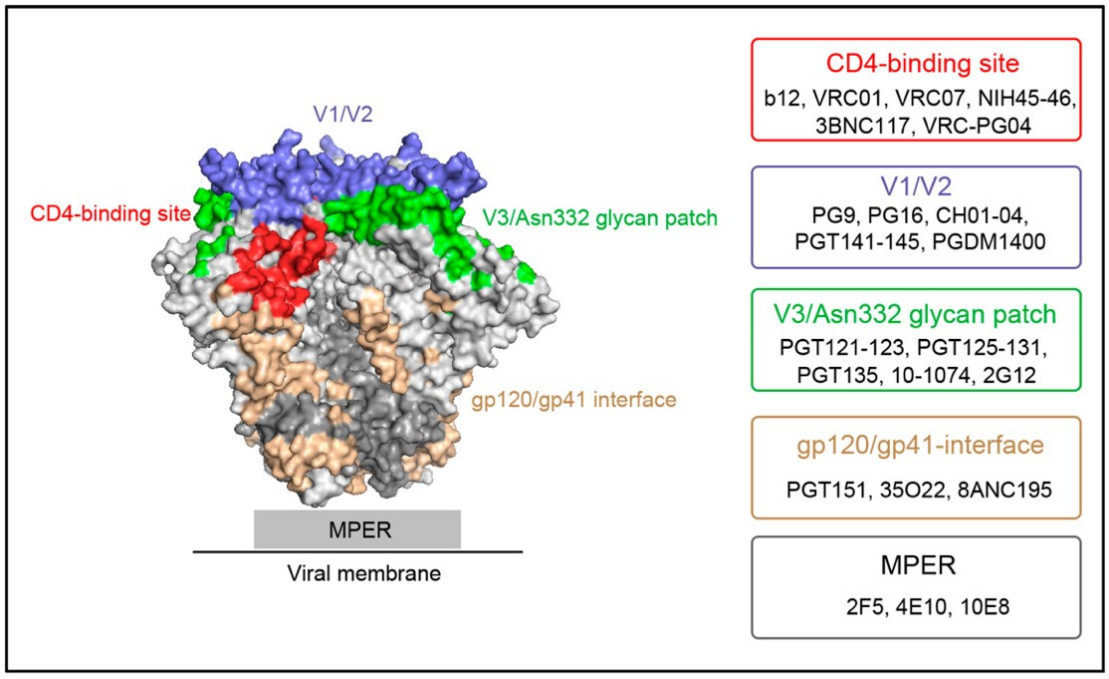
Epitopes for bNAbs on the HIV-1 envelope glycoprotein Env The picture shows the most common epitopes on the surface of an Env trimer, that are recognized by different bNAb classes which are specified in the boxes on the right. The CD4-binding site is marked red, V1/V2 blue, V3/Asn322 glycan patch green, gp120/gp41-interface brown and Membrane Proximal External Region (MPER) is represented by the dark grey box. Other surface areas of Env are colored in grey: light grey representing parts belonging to gp120, and dark grey representing parts belonging to gp41. Adapted from the references^46,47^(distributed under the “Creative Commons Attribution (CC-BY) license”).

The most common epitopes for bNAbs on the HIV-1 envelope glycoprotein Env are shown in Figure 4.

## 4. mRNA-based vaccines

Public interest in mRNA-based vaccines to prevent infectious diseases arose greatly during the COVID-19 pandemic. Indeed, this type of vaccine has shown to be highly effective against SARS-CoV-2 in the clinical trials and helped in containing morbidity and mortality by immunizing vaccinated people around the world^48-51^. Other advantages of this vaccine design method include the safety of the use of mRNA vaccines, the feasibility of large-scale production, and the high adaptability of these vaccines to combat various diseases.

### 4.1 What do mRNA vaccines consist of?

mRNA-based vaccines consist of a single-stranded mRNA encoding a specific protein of the viral particle that the immune system should be educated to recognize^52,53^. UTR sequences, a 5’ cap, a 3’ poly(A) tail flank the coding region and nucleosides within the mRNA are modified in order to delay degradation in the cytoplasm, decrease immunogenicity and enhance translation^52-54^. In vivo delivery of the mRNA can be achieved by transfection of the designed mRNA into antigen presenting cells (APCs), e.g. dendritic cells, that display the target antigen to the immune system when transferred back in vivo^55^. Newer methods such as the SARS-CoV-2 mRNA vaccines encapsulate the mRNA in lipid nanoparticles (LNPs) that contain cationic ionizable lipids that aid LNP-uptake into the cell^52-54^.

### 4.2 How do they work?

**Figure 5:**
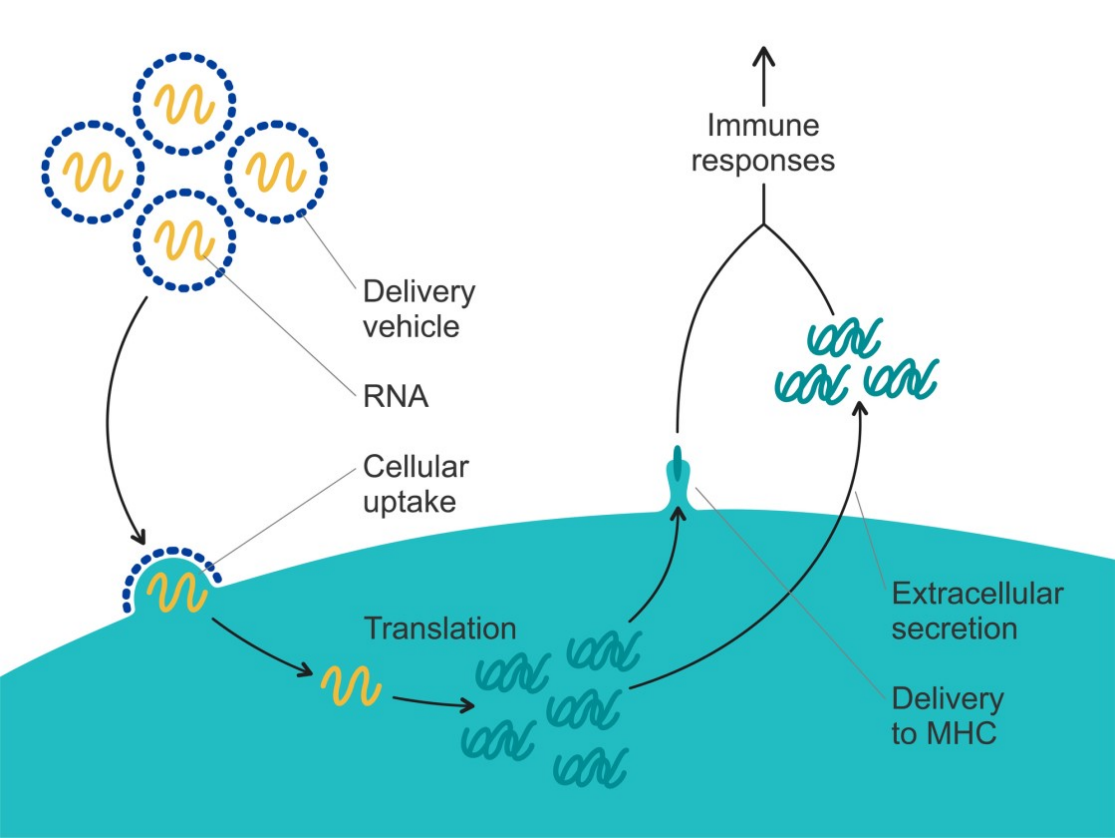
Working mechanism of mRNA-based vaccines An mRNA strand encoding the viral antigen is delivered in a LNP to facilitate cellular uptake. One inside the cell, the mRNA gets translated by the host cell’s replication machinery, so the viral protein the mRNA codes for gets produced. The viral antigen can then be displayed on MHC proteins on the cellular surface or can get secreted into the extracellular matrix, where the viral antigen is accessible for recognition by immune cells. An immune response is generated. Acquired from istockphoto.com^56^.

After delivery into a host cell, the mRNA gets translated into the viral protein using the host cell’s translation machinery^54,55^. Thus, a normal body cell starts producing viral proteins that can be displayed on MHC proteins on the cellular surface, where the viral protein can be detected as an antigen by the organism’s immune cells^54^. This triggers an immune response resulting in antibody production, cell-mediated apoptosis of infected body cells and the production of memory cells that remain even after the recovery process^54^. The mRNA strand gets broken down by nucleases and has a rather short half-life^54^ (see Figure 5).

### 4.3 Approaches of mRNA vaccines

There are different approaches to mRNA vaccination depending on whether the delivered mRNA replicates in the host cell or not.

#### · Self-amplifying mRNA vaccines (saRNA)

Besides the gene for the target antigen, this type of vaccine additionally contains genes that encode an RNA-dependent RNA polymerase^57^. These genes are obtained from RNA viruses, mostly alphaviruses^57,58^. Thus, saRNAs are able to multiply their mRNA in the host cell which results in an increased production of the encoded immunogen^57^. Although already very small doses of vaccine have shown to produce a strong and potent immune response, problems regarding delivery of the large saRNA size and higher risk of reactogenicity have to be faced^57,58^.

#### · Non-replicating mRNA vaccines

Currently, this type of vaccine is used for the containment of the SARS-CoV-2 pandemic^54^. It contains the information for synthesis of the antigen of interest as described above. In comparison to saRNA, non-replicating mRNA vaccines cannot multiply in the host cell^57^. Hence, the generated immune response is weaker but still potent^54,57^.

#### · mRNA vaccines for cancer

mRNA vaccines cannot only be used for infectious diseases, but have great potential in combating cancers by teaching the immune system which tumor antigens it has to look for^54,58^. Due to the mutations that occur when cells become cancerous, cancer cells often display so-called neoantigens with neoepitopes^58^. When analyzing tumors, such neoepitopes can be used to design an mRNA vaccine^58^. The aim is to activate an immune response in order to eliminate cancerous cells^58^. At the moment, there are many studies exploring the use of mRNA vaccines in various cancers^58^.

## 5. mRNA-based vaccines for HIV

Several studies have been conducted investigating different mRNA vaccine approaches against HIV and research is ongoing^34,59-68^. In Table 1, some of the recent promising results are presented. The first phase 1 clinical trials have been launched to evaluate the safety and immunogenicity of different HIV mRNA vaccine formulas (Table 2).

**Table 1:** Recent studies investigating different HIV-mRNA vaccine approaches

STUDY	YEAR	DESCRIPTION
Pardi et al.^64^	2017	In their study, Pardi et al. injected mice with LNP-encapsulated nucleoside-modified mRNA encoding the light and heavy chain of VRC01, a bNAb against HIV. VRC01 could be detected in the blood and generated immunity against intravenously injected HIV-1 viruses.
Pardi et al.﻿^65^	2019	The same group later used a nucleoside-modified and purified mRNA-LNP vaccine that encoded a clade C Env protein. This vaccine was injected intradermally into rabbits and rhesus macaques and caused the production of antibodies against gp120, while ADCC was also observed.
Bogers et al.^66^	2015	This group encoded a clade C Env protein into a saRNA, used cationic nanoemulsion (CNE) for delivery and injected the formula into rhesus macaques. Activation of the immune system by the synthesized viral particles resulted in strong humoral and cell-mediated immune responses, already from a low dosis.
Movo et al.^67^	2019	They induced a saRNA containing tHIVconsvX, a mix of six highly conserved HIV immunogens, into mice. Results indicated strong CD4+ and CD8+ immune responses that lasted 22 weeks after injection.
Zhang et al.^34,68^	2021	Combining several strategies, this recent study used an mRNA-based vaccine. Firstly, experiments were conducted on mice, injecting them with an mRNA vaccine encoding the Env and Gag protein. After endogenous protein synthesis in the host cell, these proteins assemble to build virus-like proteins (VLPs) that are immunogenic but not infectious as they lack the viral genome encoding the instructions for replication. An important advance in this mRNA vaccine is the similarity to natural Env protein and the quantity of the Env proteins generated on the VLPs that induced antibody production in the mice. Secondly, macaques were used to further investigate the results of this env-gag VLP mRNA vaccine. In a first step, the macaques were primed with an mRNA encoding clade B Env protein that has been modified in a way to be easily recognized by the immune system. Then, the animals received multiple env-gag boosters over one year, each consisting of gag-mRNA and a changing env-mRNA. In total, four different env-mRNAs from three HIV-1 clades were used, because antibody production should be directed towards eliciting antibodies (bNAbs) against the more conserved areas of the Env protein. The vaccine induced production of neutralizing antibodies and T-cell responses, while causing only mild adverse effects in the macaques, e.g. appetite loss. Thirdly, the protection of immunized animals compared to a control group was assessed from week 60 by 13 weekly injections of simian-human immunodeficiency virus (SHIV) into the rectum. Out of 7 animals, 2 remained uninfected, whereas the others showed an infection delay of on average 5 weeks compared to the control group. Thus, the risk of infection with SHIV per exposure was reduced by 79% in the vaccinated rhesus macaques.

**Table 2:** HIV-mRNA vaccines currently undergoing clinical trial

Clinical trial identifiers	Vaccine	Study Sponsor	Collaborators	Status	Participants
NCT05217641 HVTN302^59,60^	BG505 MD39.3 mRNA, BG505 MD39.3 gp151 mRNA, BG505 MD39.3 gp151 CD4KO mRNA	National Institute of Allergy and Infectious Diseases (NIAID)	Scripps Research Institute; Bill and Melinda Gates Foundation; Cambridge University; ModernaTX, Inc.; 11 study sites in the US	Phase 1	108 estimated participants healthy, HIV-negative ages 18 - 55
NCT05001373 IAVIG002^61,62^	eOD-GT8 60mer mRNA Vaccine (mRNA-1644) Core-g28v2 60mer mRNA Vaccine (mRNA-1644v2-Core)	International AIDS Vaccine Initiative (IAVI)	ModernaTX, Inc.; Scripps Research Institute; 4 study sites (in Atlanta, Seattle, San Antonio)	Phase 1	56 estimated participants healthy, HIV-negative ages 18 - 50

One clinical trial has been launched by a division of the US National Institute of Health (NIH)^59,60^, and another clinical trial is funded by the National Institute of Allergy and Infectious Diseases (IAVI)^61,62^, representing some of the cases where the mRNA technology is for the first time employed as an anti-HIV strategy. The mRNA HIV vaccines in both studies have been developed in cooperation with Moderna (Cambridge, MA, USA).

The HVTN302 (NCT05217641) study is based on previous work of Steichen et al.^63^. This study group designed HIV Env trimers with germline-targeting mutations that induced priming PGT121-antibody responses in mice. They further proposed a vaccine containing BG505 MD39.3 variants administered in booster injections to enhance PGT121-specific-bNAb production by guiding the affinity maturation of precursor B cells. Currently, this approach is being tested in the clinical trial HVTN302 using mRNA-based delivery^59,60^. Healthy participants are injected with one of the three mRNA vaccines intramuscularly at doses of either 100µg or 250µg and will receive a booster vaccination at month two and six. Participants' general and immune response to the vaccine will be evaluated in blood and lymph-node fine-needle aspiration probes. The higher dose will only be tested if the lower dose raises no safety concerns.

Building on the promising results of the eOD-GT8 60mer vaccine (NCT03547245), the clinical trial IAVIG002 aims to assess the safety and efficacy of the eOD-GT8 nanoparticles for germline-targeting using an mRNA platform^43,61,62^. Healthy participants will be injected with mRNA-1644 first and mRNA-1644v2-Core sequentially to stimulate precursor B cells into producing bNAbs. In other arms of the study, participants will be injected with mRNA-1644 or mRNA-1644v2-Core only.

This article focuses on mRNA vaccine technologies for active immunization against HIV. Nevertheless, it is worth mentioning that mRNA-based vaccines are currently also being tested in HIV-infected patients with the goal of passive immunization. Therefore, dendritic cell (DC) mRNA vaccines are mainly being used^55^. To do so, autologous DCs are either transfected with antigen-encoding mRNA *ex vivo* and then reinjected intradermally into the patient or the DCs are being targeted with the mRNA *in situ* by intranodal injection^55^. Several clinical trials have already been conducted investigating these strategies and others are ongoing^55^.

## 6. Benefits and limitations of the anti-HIV mRNA-based vaccines

mRNA-based vaccines in general and anti-HIV mRNA-based vaccines in particular have several significant benefits compared with conventional vaccines, in terms of safety, efficacy, production and applications (see Table 3). As limitations, mRNA-based vaccines are based on a relatively new technology and the adverse effects on long term are yet to be determined.

**Table 3:** Benefits and Limitations of anti-HIV mRNA-based vaccines

BENEFITS & LIMITATIONS	COMMENTS
Relatively new technology	Although research for mRNA-based vaccine development started decades ago, the worldwide use of mRNA vaccines has only begun recently, in the search for a potent anti-SARS-CoV-2 vaccine^54,69^. Adapting the mRNA platform to act against HIV is a very promising approach^55^. However, besides the major challenges encountered trying to find a safe and potent HIV-immunogen to be encoded within the mRNA, new anti-HIV vaccines require thorough evaluation to ensure they fulfill their function and are safe to use in the population^55^.
Efficacy	Studies as well as the COVID-19 pandemic have confirmed the effectiveness of mRNA vaccines^48,49,54,70^. The mRNA intake is facilitated by LNPs, which enables body cells to produce the viral antigen, so a strong immune response can be generated and provides the organism immunity^54^. However, the complex characteristics of HIV pose a great challenge to potent HIV vaccine design and it still remains to be determined whether the current approaches reach their intended effect^55^.
Safety	mRNA vaccines are not infectious and there is no ground for concerns that it could integrate into the host genome^54,58,70^. Due to its short in vivo half-life the mRNA strand is degraded quickly and is thus less likely to cause any side effects because of immunogenicity^54,70^. However, concerns about safety of the anti-HIV vaccine may arise regarding the immune system’s reaction to the Env protein encoded in the mRNA sequence and the possible short- and long-term adverse effects^55^.
Production	Compared with traditional forms of vaccination, less resources are needed for mRNA vaccine production. Firstly, this refers to the time needed: no viruses need to be grown in order to obtain the needed viral particle^54,70^. Secondly, material can be saved because tissues for viral growth become redundant54. Thirdly, costs can be saved, with the exception of costs that incur for distribution and storage of mRNA vaccine vials at low temperatures^54^.
Distribution and Storage	The downside of mRNA-based vaccines lies in the challenge of maintaining the low temperatures required for mRNA-vaccine storage during vaccine transport and distribution. In addition to the special cooling equipment, a well-organized logistic network is needed to ensure quality control following the guidelines for temperatures and shelf-life during shipping and storage^54,71^. Thus, the risk of faults occurring during transport might pose an obstacle, especially in low-income countries^72^.
Potential Applications	mRNA vaccines are highly adaptable, as the same platform can be used to fight various diseases. Once the viral genome is known and a section encoding a specific protein is selected, vaccine production can start at a large scale^54,58^. This feature makes mRNA vaccines a very potent instrument in containing emerging pandemics^54,70,73^.

## 7. Conclusion

To present, there are no effective agents that can be used for HIV prevention. Currently, the only method to contain the pandemic is through education by raising awareness about the disease. During the last years, a lot of research has been conducted for developing a potent HIV vaccine. Major challenges include huge variability of HIV antigens in the viral envelope and the frequent escape mutations occuring in the virus. Because of their ability to bind more conserved epitopes of the Env protein of HIV, bNAbs provide a useful source for immunization efforts. However, directing B-cell-precursors into producing bNAbs is difficult because their maturation process is complex and targeting those conserved epitopes proved to be complicated due to the hidden nature of those epitopes within the Env antigen.

Since the beginning of the SARS-CoV-2 pandemic, the usage of mRNA to deliver instructions for protein synthesis of target proteins into host cells has been greatly promoted. mRNA vaccines are safe and efficient, but also allow for large-scale manufacturing to facilitate accessibility to the vaccine worldwide. One of the greatest advantages, however, is the possibility of adapting mRNA vaccines to fight various diseases, simply by replacing the sequence in the mRNA used to code for a different target protein.

Thus, this type of vaccine is appealing for HIV vaccine research. Indeed, there have been several studies using mRNA-based vaccines to pursue and combine different strategies to induce bNAb production in the host cells that could be the basis for HIV immunity. Results were very promising and the first clinical trials evaluating mRNA-based HIV vaccines are ongoing, but a lot of research is still needed to assess and further improve the HIV-mRNA vaccination. Hence, until we will find a potent HIV vaccine that can change the course of this pandemic, obstacles still have to be overcome. Still, the most recent findings are encouraging and call for more research to pursue this goal of finding a vaccine against HIV.

## KEY POINTS

**◊** *Human immunodeficiency virus (HIV), the cause of AIDS, poses a serious health issue around the world*

**◊** *Until now, most approaches have focused on the use of bNAbs for development of an HIV vaccine. Lately, mRNA-based HIV vaccines have emerged as a promising method*

**◊** *HIV presents a difficult target for vaccine production because of its high variability in envelope protein structure and mutation rate*

**◊** *Currently, the first mRNA-based HIV vaccines are being tested in clinical trials*
